# Genetic analysis of limbic-predominant age-related TDP-43 encephalopathy neuropathologic change in a population-based cohort of the oldest old

**DOI:** 10.1093/braincomms/fcag189

**Published:** 2026-05-28

**Authors:** Elizaveta Mikhailenko, Sara Savola, Mia Kero, Pentti J Tienari, Liisa Myllykangas, Karri Kaivola

**Affiliations:** Department of Pathology, University of Helsinki, Helsinki 00014, Finland; Department of Pathology, HUS Diagnostic Center, Helsinki University Hospital, Helsinki 00029, Finland; Department of Pathology, University of Helsinki, Helsinki 00014, Finland; Department of Pathology, HUS Diagnostic Center, Helsinki University Hospital, Helsinki 00029, Finland; Department of Pathology, University of Helsinki, Helsinki 00014, Finland; Department of Pathology, HUS Diagnostic Center, Helsinki University Hospital, Helsinki 00029, Finland; Translational Immunology, Research Programs Unit, University of Helsinki, Helsinki 00014, Finland; Department of Neurology, University of Helsinki and Helsinki University Hospital, Helsinki 00014, Finland; Department of Pathology, University of Helsinki, Helsinki 00014, Finland; Department of Pathology, HUS Diagnostic Center, Helsinki University Hospital, Helsinki 00029, Finland; Translational Immunology, Research Programs Unit, University of Helsinki, Helsinki 00014, Finland; Department of Neurology, University of Helsinki and Helsinki University Hospital, Helsinki 00014, Finland

**Keywords:** neuropathology, limbic-predominant age-related tdp-43 encephalopathy, genetics, protein tdp-43, mixed pathology

## Abstract

Limbic-predominant age-related TDP-43 encephalopathy neuropathologic change is a common proteinopathy in the oldest old that is associated with cognitive decline. Although the genetic basis of limbic-predominant age-related TDP-43 encephalopathy neuropathologic change remains largely unknown, *TMEM106B*, *GRN* and *APOE* loci are frequently implicated. Here, we examined nine previously reported limbic-predominant age-related TDP-43 encephalopathy neuropathologic change risk loci (*ARHGEF28, APOE, GRN, KAZN, LHX1, TPCN1, TMEM106B, UNC13C* and *WWOX*) in a population cohort of 262 individuals from the Vantaa 85 + study. We also tested whether Alzheimer’s disease polygenic risk score without *APOE* was associated with limbic-predominant age-related TDP-43 encephalopathy neuropathologic change. Using ordinal logistic regression models, *GRN* rs5848 (odds ratio = 2.45, 95% confidence interval: 1.71–3.52, adjusted *P* = 5.75 × 10^−6^), *APOE* ε4 dose (odds ratio = 1.73, 95% confidence interval: 1.07–2.80, adjusted *P* = 0.030) and *KAZN* rs72643142 (odds ratio = 2.38, 95% confidence interval: 1.38–4.11, adjusted *P* = 0.0048) were associated with higher limbic-predominant age-related TDP-43 encephalopathy neuropathologic change stage. Additionally, Alzheimer’s disease polygenic risk score without *APOE* was associated with limbic-predominant age-related TDP-43 encephalopathy neuropathologic change after adjusting for age, sex, Alzheimer’s disease pathology and *APOE* ε4 dose (odds ratio = 1.36, 95% confidence interval: 1.06–1.75, adjusted *P* = 0.027). Our findings contribute to the understanding of limbic-predominant age-related TDP-43 encephalopathy neuropathologic change genetics and suggest shared biological processes between limbic-predominant age-related TDP-43 encephalopathy neuropathologic change and Alzheimer’s disease.

## Teaser

Mikhailenko *et al*. report that *GRN*, *APOE* and *KAZN* loci are associated with limbic-predominant age-related TDP-43 encephalopathy neuropathologic change in a population-based cohort of the oldest old. Alzheimer’s disease polygenic risk without *APOE* is independently associated with disease severity, supporting a potential link between Alzheimer’s pathology and TDP-43 proteinopathy.

## Graphical Abstract

**Graphical Abstract:**
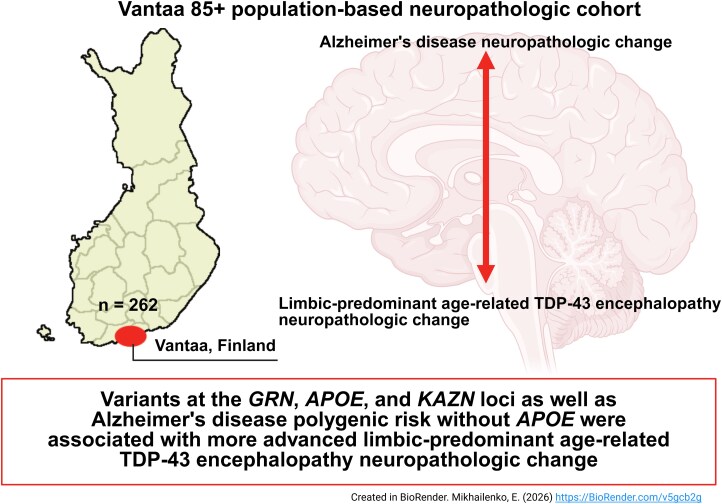

## Introduction

Limbic-predominant age-related TDP-43 encephalopathy neuropathologic change (LATE-NC) is a neurodegenerative condition characterized by the accumulation of TDP-43 protein in the amygdala, hippocampus and/or frontal cortex.^1^ LATE-NC is present in more than 30% of people over 85 years,^2^ and it is independently associated with cognitive decline.^3,4^ LATE-NC may also worsen the effects of *APOE* ε4 on cognition, independent of Alzheimer’s disease pathology.^5,6^ Moreover, it was recently reported that LATE-NC is causally related and likely the predominant cause of hippocampal sclerosis of ageing.^7^ Despite the high prevalence and detrimental effects LATE-NC has on cognition, the aetiology of LATE-NC remains largely unknown. Unravelling the pathological processes of LATE-NC is crucial to elucidate the risk factors and biological pathways that drive this late-onset proteinopathy.

To date, only one genome-wide association study has been performed on a specific LATE-NC phenotype.^8^ In this study, *TMEM106B* and *APOE* were the only genome-wide significant loci, with *GRN* and *LHX1* showing suggestive associations. The associations of *APOE, TMEM106B* and *GRN* with LATE-NC or TDP-43 accumulation had been previously identified and replicated in smaller studies,^9-11^ but *LHX1* was a novel locus. In addition to the replicated LATE-NC loci, several candidate loci have been previously reported. Variants in *WWOX, ABCC9* and *TPCN1* have been linked to LATE-NC,^9,10,12^ while variants in *KAZN, ARHGEF28* and *UNC13C* have been associated with a proxy factor comprising TDP-43 pathology and hippocampal sclerosis of ageing.^13^

In addition to *APOE,* another well-established Alzheimer’s disease risk locus, *SORL1,* has been associated and replicated with LATE-NC when there was coexisting Alzheimer’s disease neuropathological change.^10^ The overlap between Alzheimer’s disease and LATE-NC genetic risk loci could suggest a shared genetic aetiology or be a sign of more indirect interplay between these pathologies. Neuritic amyloid plaques could promote TDP43-proteinopathy,^1^ so the association of *SORL1,* and *APOE* ε4 with TDP-43 accumulation could be indirect and mediated by amyloid pathology. It has also been reported that the aetiology between ‘pure’ LATE-NC and LATE-NC with moderate to severe Alzheimer’s disease neuropathologic change could differ.^14^

Here, we studied the association of previously reported loci with LATE-NC in the population-based Vantaa 85+ study. There have been only few studies that have assessed the genetic risk factors of LATE-NC in a population-based setting. Our study enables the exploration of previously reported LATE-NC genetic associations in a single, uniformly assessed neuropathological population cohort, thereby refining the understanding of the genetic aetiology of LATE-NC. Additionally, we investigated whether an overall Alzheimer’s disease genetic risk is associated with LATE-NC, or if the association is primarily *APOE*-related.

## Materials and methods

### Study cohort and histological analyses

The cohort description and histological analyses, including the assessment of TDP-43 pathology, have been previously reported.^3,15^ In brief, the Vantaa 85+ study comprises all individuals aged 85 years or older who were residents of the city of Vantaa, Finland, as of April 1, 1991 (*n* = 601). Of the whole cohort, 304 underwent autopsy. We used a rabbit polyclonal anti-C-terminal TDP-43 antibody (Proteintech, concentration 0.3 mg/ml) with LabVision immunostainers and the Agilent Dako EnVision FLEX+ detection system (K8002 kit with rabbit linker K8009) to visualize TDP-43 pathology. This antibody was chosen because it works well in a long-fixed material.^3^ We used TDP-43-positive neurofibrillary tangle-like and granular neuronal cytoplasmic inclusions (NCI) and TDP-43-positive processes to stage LATE-NC. Stage 1a represented cases with TDP-43-positive NCI only in the amygdala. Stage 1b represented cases with TDP-43-positive NCIs that were limited to the hippocampus. For stage 1c, which includes cases with TDP-43-positive cell processes but without NCI in amygdala and/or hippocampus, we accepted cases that showed at least three TDP-43-positive processes in either amygdala or hippocampus in the absence of NCI. In stage 2, there were TDP-43-positive NCI in both amygdala and hippocampal sections, and stage 3 cases had TDP-43-positive NCI in the amygdala, hippocampus and middle frontal gyrus.

### Genotyping and imputation

The Vantaa 85+ samples were genotyped with Illumina Infinium Human370 BeadChips (Illumina, San Diego, CA, USA). The genotype data were merged with a larger dataset originating from a similar genotyping approach and pre-phased with Eagle 2.3.5 (https://data.broadinstitute.org/alkesgroup/Eagle/) using the default parameters, except that the number of conditioning haplotypes was set to 20 000.

Genome-wide genotype imputation was performed with the population-specific SISu v3 imputation reference panel using Beagle 4.1 (version 27Jan18.7e1, https://faculty.washington.edu/browning/beagle/b4_1.html) following a previously published protocol (https://dx.doi.org/10.17504/protocols.io.xbgfijw). In post-imputation QC, variants with an imputation INFO score < 0.7 or a minor allele frequency < 0.01 were removed. We extracted nine variants that were previously associated with LATE-NC from the imputed data: *TMEM106B* rs13237518, *APOE* genotype (based on rs429358 and rs7412), *GRN* rs5848, *LHX1* rs11867450, *WWOX* rs6564590, *TPCN1* rs6489896, *KAZN* rs72643142, *UNC13C* rs141108370 and *ARHGEF28* rs80190672. The *SORL1* variant rs74685827 was too rare to be included in this study. As a supplementary analysis, we also tested the association between LATE-NC and HS-associated loci (Supplementary Table 1).

To study the linkage disequilibrium between the *TMEM106B* variant rs13237518 and the *TMEM106B* AluYb8 element,^16^ we used Manta^17^ with default parameters at the *TMEM106B* locus to determine the Alu element variant from previously generated whole-genome sequencing data of 282 Vantaa85+ participants.^18^

### Polygenic risk scores

We selected an Alzheimer’s disease polygenic risk score without *APOE* that was built and validated in European cohorts^19^ via the PGScatalog^20,21^ (PGS000898). This polygenic risk score consisted of 39 variants, of which 35 were present in our data. We used PLINK2^22^ to perform linear scoring with default parameters. We calculated z-scores based on the whole imputed and quality-controlled population-based cohort of 504 samples.

### Statistical analysis

We conducted a descriptive Fisher’s exact tests in R (version 4.2.0) to evaluate whether allele frequencies of the candidate genes’ variants significantly differed across any LATE-NC stages in a 2 × 6 contingency table. The results for *APOE* and *TMEM106B* have been previously reported.^3^ We used ordinal logistic regression modelling as the main analysis. Ordinal logistic regression models were built for variants with *P* < 0.05 in the Fisher’s test. Ordinal logistic regression models were performed as implemented in the MASS package in R, adjusting for age and sex. We built the ordinal logistic regression models separately for each risk variant. The ordinal regression model investigating Alzheimer’s disease polygenic risk score without *APOE* was also adjusted for *APOE* ε4 dose and a binary variable on AD pathology (NIA-AA ABC score ‘Not’/’Low’ versus ‘Intermediate’/High’). In the ordinal logistic regression modelling, we regrouped the original LATE-NC stages into four levels, adapting the levels used in the LATE-NC GWAS analysis.^8^ We excluded the LATE-NC stage 1c from ordinal logistic regression models as it was not included in the original LATE-NC consensus recommendations. Moreover, LATE-NC stage 1c was not associated with cognitive decline in our previous study^3^ and its significance still remains uncertain. The levels were:

Level 0: No recorded TDP-43 NCI (LATE-NC stage 0).

Level 1: TDP-43 deposits restricted to the amygdala or hippocampal region (LATE-NC stages 1a and 1b).

Level 2: TDP-43 deposits present in both the amygdala and hippocampal region (LATE-NC stage 2).

Level 3: TDP-43 deposits affecting the neocortex, hippocampal formation and amygdala (LATE stage 3).

We controlled the false discovery rate using the Benjamini–Hochberg procedure. *P*-values from the ordinal logistic regression models were ranked from smallest to largest, and statistical significance was determined using a family-wise threshold of α = 0.05.

## Ethical approval

The ethical committee of the city of Vantaa and the Helsinki University Hospital have approved the study. The Finnish Health and Social Ministry permitted the assessment of death certificates and health and social work records for research. The National Authority for Medicolegal Affairs (Valvira) approved collecting the tissue samples and their research use. Each participant and/or their relatives gave informed consent for the study.

## Results

### Candidate-gene analysis

After quality control, we analysed the association of eight variants at the *TMEM106B*, *GRN*, *KAZN*, *ARHGEF28*, *UNC13C*, *LHX1*, *WWOX* and *TPCN1* loci and *APOE* ε4 dose with LATE-NC in 262 participants of the Vantaa85+ study (86% female, mean age 92.6 years). The *SORL1* variant rs74685827 was too rare to be included in this study.

Out of the nine genetic variants, *TMEM106B* rs13237518, *GRN* rs5848, *APOE* ε4 and *KAZN* rs72643142 had allele frequencies that differed between LATE-NC stages (Fisher’s exact test *P* < 0.05, Table 1).

**Table 1:** Allele counts of variants previously associated with LATE-NC

Variant	Stage 0	Stage 1a	Stage 1b	Stage 1c	Stage 2	Stage 3	*P*-value
***APOE* ε4**							**0**.**028**
No ε4 allele	162 (89.0%)	40 (74.1%)	22 (78.6%)	42 (91.3%)	117 (79.1%)	56 (84.8%)	
One or two ε4 alleles	20 (11.0%)	14 (25.9%)	6 (21.4%)	4 (8.7%)	31 (20.9%)	10 (15.2%)	
**rs80190672 (*ARHGEF28*)**							0.58
A-allele	172 (94.5%)	48 (88.9%)	26 (92.3%)	44 (95.7%)	140 (94.6%)	60 (90.9%)	
G-allele	10 (5.5%)	6 (11.1%)	2 (7.1%)	2 (4.3%)	8 (5.4%)	6 (9.1%)	
**rs5848 (*GRN*)**							**3.3** **×** **10^−5^**
C-allele	142 (78.0%)	38 (70.4%)	19 (67.9%)	35 (76.1%)	94 (63.5%)	29 (43.9%)	
T-allele	40 (22.0%)	16 (29.6%)	9 (32.1%)	11 (23.9%)	54 (36.5%)	37 (56.1%)	
**rs72643142 (*KAZN*)**							**0**.**019**
C-allele	174 (95.6%)	50 (92.6%)	25 (89.3%)	44 (95.7%)	127 (85.8%)	57 (86.4%)	
T-allele	8 (4.4%)	4 (7.4%)	3 (10.7%)	2 (4.3%)	21 (14.2%)	9 (13.6%)	
**rs11867450 (*LHX1*)**							0.22
G-allele	109 (59.9%)	36 (66.7%)	20 (71.4%)	36 (78.3%)	92 (62.2%)	39 (59.1%)	
A-allele	73 (40.1%)	18 (33.3%)	8 (28.6%)	10 (21.7%)	56 (37.8%)	27 (40.9%)	
**rs13237518 (*TMEM106B*)**							**0**.**025**
C-allele	121 (66.5%)	33 (61.1%)	22 (78.6%)	25 (54.3%)	98 (66.2%)	54 (81.8%)	
A-allele	61 (33.5%)	21 (38.9%)	6 (21.4%)	21 (45.7%)	50 (33.8%)	12 (18.2%)	
**rs6489896 (*TPCN1*)**							0.54
T-allele	154 (84.6%)	47 (87.0%)	23 (82.1%)	40 (87.0%)	132 (89.2%)	61 (92.4%)	
C-allele	28 (15.4%)	7 (13.0%)	5 (17.9%)	6 (13.0%)	16 (10.8%)	5 (7.6%)	
**rs141108370 (*UNC13C*)**							0.16
A-allele	180 (98.9%)	52 (96.3%)	26 (92.3%)	45 (97.8%)	145 (98.0%)	66 (100.0%)	
G-allele	2 (1.1%)	2 (3.7%)	2 (7.1%)	1 (2.2%)	3 (2.0%)	0	
**rs6564590 (*WWOX*)**							0.45
C-allele	117 (64.3%)	32 (59.3%)	16 (57.1%)	32 (69.6%)	101 (68.2%)	37 (56.1%)	
G-allele	65 (35.7%)	22 (40.7%)	12 (42.9%)	14 (30.4 %)	47 (31.8%)	29 (43.9%)	

*N* = 262 (42 autopsied individuals excluded due to missing genetic data or TDP-43 data). *P*-values < 0.05 are bolded.

*P*-value, raw *P*-value from Fisher’s exact test on a 2 × 6 contingency table.

We further analysed the association of *TMEM106B* rs13237518, *GRN* rs5848, *APOE* ε4 and *KAZN* rs72643142 variants using ordinal logistic regression. *GRN* rs5848 strongly associated with increasing LATE-NC stage [odds ratio (OR) = 2.45, 95% confidence interval (CI): 1.71–3.52, adjusted *P* = 5.75 × 10^−6^], as did *APOE* ε4 dose (OR = 1.73, 95% CI:1.07–2.80, adjusted *P* = 0.030). We observed a significant association for *KAZN* rs72643142 (OR = 2.38, 95% CI: 1.38–4.11, adjusted *P* = 0.0048). *TMEM106B* rs13237518 showed a protective effect (OR = 0.72, 95% CI: 0.49–1.05), but the association was not statistically significant (adjusted *P* = 0.091, Table 2). The direction of the effects corresponded to previous reports, except for *KAZN* rs72643142.

**Table 2:** Ordinal logistic regression on LATE-NC

	Effect allele	Other allele	OR (95% CI)	*P*-value	Adjusted *P*-value
**rs5848 (*GRN*)**	T	C	2.45 (1.71–3.52)	1.15 × 10^-6^	5.75 × 10^-6^
**rs72643142 (*KAZN*)**	T	C	2.38 (1.38–4.11)	0.0019	0.0048
**AD PRS without *APOE***	NA	NA	1.36 (1.06–1.75)	0.016	0.027
***APOE***	ɛ4	ɛ3/ɛ2	1.73 (1.07–2.80)	0.024	0.030
**rs13237518 (*TMEM106B*)**	A	C	0.72 (0.49–1.05)	0.091	0.091

Ordinal logistic regression models were adjusted for sex and age of death. The model with AD PRS without *APOE* was also adjusted with *APOE ε4* dose and Alzheimer’s disease pathology. *N* = 239 (23 individuals with LATE-NC stage 1c excluded).

*P*-value, raw *P*-value from ordinal logistic regression run on a four-level LATE-NC stage variable: stage 0 – stage 1a+stage 1b – stage 2 – stage 3; Adjusted *P*-value, Benjamini–Hochberg adjusted *P*-value; AD PRS, Alzheimer’s disease polygenic risk score; NA, not applicable.

As a supplementary analysis, we studied twelve variants previously associated with hippocampal sclerosis of ageing in LATE-NC, but no variant reached statistical significance (Supplementary Table 1).

### Assessment of the *TMEM106B* Alu element in the *TMEM106B* risk haplotype

An AluYb8 element has been suggested to be a potential causative variant at the *TMEM106B* locus.^16^ In 299 whole-genome-sequenced samples, we identified a 322 bp deletion (chr7:12242077-12242077, hg38) whose sequence matched that of the Alu element. The genotypic concordance of the *TMEM106B* variant rs13237518-A and *TMEM106B* Alu element deletion was very high (289/299, 96.7%). This confirmed that the Alu element is a part of the risk haplotype also in the Finnish population, but it was not possible to tease apart its effect from *TMEM106B* variant rs13237518.

### LATE-NC and Alzheimer’s disease polygenic risk score without *APOE*

*APOE* ε4 has commonly associated with LATE-NC^8-11,13^ but Alzheimer’s disease pathology and LATE-NC commonly coexist.^11^ We investigated their interdependence by examining whether a previously validated Alzheimer’s disease polygenic risk score that excludes *APOE*^19^ is associated with LATE-NC.

In an ordinal logistic regression model adjusted for age, sex, *APOE* ε4 dose and a dichotomous variable on Alzheimer’s disease pathology, Alzheimer’s disease polygenic risk score without *APOE* was associated with an increasing LATE-NC stage (OR = 1.36, 95% CI: 1.06–1.75, adjusted *P* = 0.027, Table 2).

## Discussion

Previously, there have been very few studies focused on genetics of LATE-NC in a population-based setting. In this study, we performed a candidate-gene analysis of LATE-NC in a well-characterized population-based neuropathology cohort of the oldest old. Among previously unreplicated loci, *KAZN* showed a statistically significant association with LATE-NC in our data. Moreover, we found that Alzheimer’s disease polygenic risk score without *APOE* associated with LATE-NC.

Our findings reinforce prior evidence on *GRN*. In previous studies, *TMEM106B* variants have been more robustly associated with both LATE-NC and hippocampal sclerosis of ageing^8,9^ than *GRN* variants. In our cohort, however, *GRN’s* absolute effect size was roughly 1.5 times higher than *TMEM106B’s*. This discrepancy may reflect cohort- or population-specific effects, differences in case ascertainment, or underlying heterogeneity in genetic susceptibility. Of note, *TMEM106B rs13237518* allele T mostly protected against LATE-NC stage 3 in our data (Table 1). Consequently, *TMEM106B rs13237518* did not show statistically significant association with the ordinal LATE-NC variable after adjustment for age at death, sex and multiple testing correction (OR = 0.72, 95% CI: 0.49–1.05, *P* = 0.091). In the genome-wide association study on LATE-NC with a similar ordinal logistic regression model, the effect size of the *TMEM106B* locus (OR = 0.70, 95% CI: 0.63–0.78) was almost exactly the same as in our data. This could suggest our study was underpowered to observe the effect of the *TMEM106B* locus. Another potential explanation could be that the *TMEM106B* locus is truly mostly protective against LATE-NC stage 3, and the effect is therefore not as evident in a population-based cohort where LATE-NC stage 3 might not be as common as in other study types.

It was recently suggested that the functional variant at the *TMEM106B* risk locus could be an AluYb8 element in the 3′ untranslated region of the *TMEM106B* gene.^16^ In our data, the AluYb8 element deletion was present in 96.7% of the *TMEM106B* risk haplotypes. This confirms the linkage of the AluYb8 element and the risk haplotype in individuals with Finnish ancestry. However, due to the high linkage disequilibrium, it was not possible to pinpoint the aetiological variant on the risk haplotype.

The only previously unreplicated locus that showed a statistically significant association with LATE-NC in our data was the *KAZN* locus. The *KAZN* locus was previously associated with a decrease in TDP-43 factor score and hippocampal sclerosis.^13^ In our data, the *KAZN* locus increased the risk of LATE-NC, showing an inverse effect direction to the previous report. *KAZN* is highly expressed in hippocampal and cortical neurons and is involved in cell adhesion and cytoskeletal organization, suggesting plausible links to neuronal vulnerability or TDP-43 misprocessing.^23^ The inverse effect we observed could be due to, e.g. differing haplotype structures in Finnish versus other populations, variability in disease stage, or a spurious association.

As a novel finding, we also found that the association of Alzheimer’s disease genetics with LATE-NC was not limited to *APOE ε4.* Rather, Alzheimer’s disease polygenic risk score without the *APOE* locus also associated with LATE-NC. This finding likely reflects at least partly the co-occurrence of Alzheimer’s disease and LATE-NC. However, as our model was also adjusted for Alzheimer’s disease pathology, our results suggest shared biological processes between LATE-NC and Alzheimer’s disease. Alternatively to a shared genetic aetiology, it could be that Alzheimer’s disease pathology mediates the effect of Alzheimer’s disease genetics on LATE-NC. In this case, any variable with strong enough effect on Alzheimer’s disease pathology could appear associated with LATE-NC. This would also explain why the association between *SORL1* and LATE-NC has been present only when there was concomitant Alzheimer’s disease pathology.^11^

There are currently only a limited number of genetic studies of LATE-NC, and evidence supporting most proposed candidate loci remains sparse. Studying these loci in independent cohorts is therefore essential for evaluating the robustness of reported associations and for identifying factors that may influence their effects. In this context, our use of a Finnish population-based cohort allows assessment of LATE-NC genetic associations across the full disease spectrum and in a population with distinct genetic characteristics compared with the predominantly North American cohorts studied to date. Moreover, we studied individuals over 85 years of age, an age group with high prevalence of LATE-NC but often underrepresented in genetic research. In addition, we provide novel evidence that Alzheimer’s disease polygenic risk score is associated with LATE-NC independent of *APOE* ε4, highlighting a new perspective on shared or interacting genetic mechanisms underlying TDP-43 and Alzheimer’s disease pathology. However, limitations include modest sample size, restriction to Finnish ancestry, and incomplete data on certain co-pathologies (e.g. ARTAG, arteriolosclerosis). A major challenge in identifying genetic risk factors for LATE-NC lies in its frequent co-occurrence with other neuropathologies, including ADNC, vascular brain injury and alpha-synucleinopathies, which can obscure genotype-phenotype associations. Additionally, we assessed TDP-43 pathology using a C-terminal non-phosphorylated antibody as it worked well in our long-fixed tissue material.^3^ Non-phosphorylated antibodies are less sensitive than phospho-specific antibodies for detecting LATE-NC lesions. Moreover, we did not evaluate the nuclear clearance of TDP-43. Due to these limitations, some TDP-43 aggregates could have been missed and lead to incorrect LATE-NC staging. Previous comparisons, however, showed that the frequency of LATE-NC in our data was consistent with the literature, and our LATE-NC staging correlated well with clinical and other neuropathological variables.^3^

In conclusion, our findings refine the current knowledge on the genetics of LATE-NC, and elucidate the interplay of Alzheimer’s disease genetic risk and the accumulation of TDP-43. These results contribute to the growing evidence that a polygenic and multifactorial architecture shapes LATE-NC.

## Supplementary Material

fcag189_Supplementary_Data

## Data Availability

All data used and analysed in this study are available from the corresponding author upon reasonable request to the extent allowed by this study’s permits. All code used in this analysis is deposited to GitHub (https://github.com/kkaivola/LATENC).
