# Supplementary material for: Genetic analysis of limbic-predominant age-related TDP-43 encephalopathy neuropathologic change in a population-based cohort of the oldest old
Source: Brain Commun. 2026 May 28;8(3):fcag189. doi: 10.1093/braincomms/fcag189 (PMC13245399; doi:10.1093/braincomms/fcag189)
Supplement: fcag189_Supplementary_Data [file fcag189_supplementary_data.pdf]

Supplementary Table 1. Allele counts of hippocampal sclerosis-associated variants across LATE-NC stages.

	LATE-NC							
**SNV**	**Stage 0**	**Stage 1c**	**Stage 1a**	**Stage 1b**	**Stage 2**	**Stage 3**	**All participants**	**P-value**
**rs704178 (*ABCC9*)**								0.8021
C-allele	77	24	21	12	60	29	223	
G-allele	105	22	33	16	88	37	301	
**rs1914361 (*ABCC9*)**								0.4778
A-allele	84	27	24	15	67	36	253	
G-allele	98	19	30	13	81	30	271	
**rs55751884 (*WWOX*)**								0.8181
C-allele	25	6	9	3	27	12	82	
T-allele	157	40	45	25	121	54	442	
**rs9930659 (*WWOX*)**								0.7774
C-allele	85	25	25	12	64	27	238	
T-allele	97	21	29	16	84	39	286	
**rs4985556 (*IL34*)**								0.1486
A-allele	17	3	5	5	6	6	42	
C-allele	165	43	49	23	142	60	482	
**rs199515 (*MAPT*)**								0.4169
G-allele	22	4	5	3	8	5	47	
C-allele	160	42	49	25	140	61	477	
**rs871269 (*TNIP1*)**								0.06281
T-allele	69	22	16	5	59	19	190	
C-allele	113	24	38	23	89	47	334	
**rs7225151 (*SCIMP*)**								0.8653
A-allele	33	7	6	4	26	9	85	
G-allele	149	39	48	24	122	57	439	
**rs4277405 (*ACE*)**								0.5307
C-allele	59	18	22	11	43	20	173	
T-allele	123	28	32	17	105	46	351	
**rs117598708 (*USP45*)**								0.3491
G-allele	11	0	1	2	9	5	28	
A-allele	171	46	53	26	139	61	496	
**rs9792612 (*CTNNA3*)**								0.1392
T-allele	60	17	14	8	52	12	163	
C-allele	122	29	40	20	96	54	361	
**rs9927475 (*FOXF1*)**								0.9645
A-allele	38	11	12	7	34	17	119	
G-allele	144	35	42	21	114	49	405	

Calculated using Fisher's exact test
